# Surgery

**Published:** 1837-07

**Authors:** 


### SURGERY.

*Observations on Hypertrophy of the Mammary Gland.* By Dr. Fingerhuth, of Esch.

This affection of the mammæ, which depends on an enlargement of the individual acini of the gland, and an increased accumulation of fat in the cellular tissue, is characterized by a constant, uniform, and painless increase of bulk. Dr. Fingerhuth distinguishes two forms; one which runs its course rapidly, and occurs at the period of puberty as a disease of development; the other, slow in its progress, and chiefly connected with disturbance in the functions of the generative system. The following observations are limited to the illustration of the first of these species.

The swelling commences in one of the breasts, generally the right, (for both are seldom affected simultaneously,) and is ushered in by a pricking sensation in the gland, sometimes accompanied by increased sensibility. The enlargement is uniform, and involves the whole breast. The disease always makes its appearance at the period of puberty, and is contemporary with the development of the mammæ, either the patient has never menstruated, or the catamenia are scanty and soon disappear. There is, however, a feeling of increased pressure and a more rapid increase of the swelling about the period of expected menstruation, in patients who have ceased to menstruate; during the intervals, the progress of the swelling is slower, but more constant. Occasionally, the patient's voice undergoes a peculiar change; it becomes hoarse and rough: this lasts for two or more days, and then ceases for a time, appearing and disappearing without any obvious cause. In one of Dr. Fingerhuth's patients, this hoarseness occurred at the period of expected menstruation; in others, he has not noticed this alteration of the voice. When the breast has attained a more considerable size, it is superficially softer, and the hard enlarged acini cannot be felt without making deeper pressure. About this time, also, the cutaneous veins become more distended, so as to give the skin somewhat of a bluish tint; the nipple becomes flatter and broader, and the areola more extended. In this way the swelling goes on increasing with more or less rapidity, until the breast has attained a very remarkable size; in some cases it is from eighteen to twenty inches in length, and from twenty to twenty-four in circumference, and weighs from ten to twelve pounds. The perspiration and blood of the patient have a peculiar smell, and the latter contains a large quantity of free carbonic acid. As the disease proceeds, the patient becomes impeded by the weight of the organ; the rest of the body generally emaciates; the thoracic viscera begin to sympathize; the patient complains of dyspnœa and sense of weight in the chest, followed by cough, loss of strength, hectic, general exhaustion, and death. The progress of the disease, however, does not always correspond with this description, and its termination presents several varieties. Sometimes the swelling reaches a determinate height, and then remains stationary for years, or even for the patient's life, causing no other inconvenience than what is necessarily connected with its weight and bulk. Sometimes the disease terminates favorably; the swelling diminishes, and ceases to give any annoyance, but the breast never returns to the normal. Sometimes it ends in effusions of serum into sacs formed in the cellular tissue, in the interstices between the enlarged and hardened acini. Where it terminates in death, it is generally accompanied by symptoms of disease in the thoracic viscera,—as ulcerations of the bronchial mucous membrane, phthisis, and hydrothorax.

On anatomical examination, very little alteration is discoverable beyond the general increase of volume, and the enlargement of the individual acini. The cellular tissue is loose, its cells larger than natural, and containing a remarkable quantity of fat. The arteries exhibit no change, either in their structure or their caliber; but the lacteal tubes are swollen and enlarged, and the veins always remarkably dilated, and occasionally altered in their structure. The pulpy matter of the nerves seems to be diminished, and the nervous twigs appear firmer and harder than usual.

Dr. Fingerhuth looks upon this hypertrophy of the breast as a disease of development, depending on the exalted activity of the organ at the period of puberty. Among the exciting causes of the disease he reckons stimulant and heating diet, frequent manipulation, the use of fragrant washes of a stimulating nature, blows, and pressure.

With respect to the mode of treatment, he divides it into the radical and palliative. The first is accomplished by diminishing the increased activity of the organ, or by extirpating the diseased gland with the knife. The palliative treatment consists in making the patient wear a suspensory bandage, and avoid all pressure, or whatever is likely to irritate the organ; in promoting the secretions, and prescribing a light vegetable diet, gentle exercise, and cheerful recreations. The following heads of cases will serve to illustrate the nature and treatment of this affection.

Case i. L. W., aged 23, of delicate constitution. A painless swelling of the right breast made its appearance several years before, without any known cause, and gradually increased in size. When she consulted Dr. Fingerhuth, the tumour was twenty-six inches in circumference, painless, and exhibited a somewhat bluish spot at its lower part, which had a fluctuating feel. The patient was emaciated. and complained of weight about the præcordia, loss of appetite, constriction of the chest, and dry cough. She refused to submit to an operation for the removal of the diseased breast, but allowed Dr. Fingerhuth to puncture the soft part of the tumour; from which three or four ounces of yellowish serum were discharged. Compression with a bandage was next tried, but the patient could not bear it, and refused all further medical aid. She died quite exhausted about five months afterwards. On dissection, the usual phenomena of hypertrophy were discovered. At two points the acini were of a firmer and harder consistence, and between them, in the cellular tissue, were two sacs filled with yellowish serum.

Case ii. E. B., aged 17. A painless swelling of the right breast, of twelve months' standing. It had increased in a very remarkable manner during the two last months. She complained of a sense of fulness and pressure in the breast during the menstrual period; but there was no pain, and the temperature of the part was normal. She had used mercury to salivation, burnt sponge, leeches, and spirituous fomentations, without any benefit; the breast gradually enlarged, and was now double the size of the other. The swelling was uniformly soft, not tense, and capable of bearing firm pressure. The swelling suddenly increased during the menstrual period, accompanied by a sense of heat and pressure; the latter, however, ceased when menstruation was over, and the breast resumed its ordinary volume. The areola was large and somewhat dark, the nipple natural, the cutaneous veins distended, so as to give the breast a bluish tint. In other respects the patient was quite healthy.

Dr. Fingerhuth first tried the ioduret of mercury in the form of ointment: this was applied to the affected breast for twenty-four days without any benefit, and he therefore determined to have recourse to the only plan which offered a prospect of success; viz. to bring on the natural secretion of the organ. This he attempted to effect by the application of a dry cupping-glass to the affected breast. The cupping-glass was applied regularly for a fortnight, without any effect; the swelling increased, and the patient frequently complained of a feeling of heat and tension in the breast; but, on the sixteenth day, the milk began to flow in small quantity, and, at the end of the third week, a considerable quantity was discharged twice a day, and the breast became evidently diminished in size. In addition to the use of the cupping-glass, the patient was directed to take an iodine bath every fifth day, support the breast with a suspensory bandage, live on vegetable diet, and take gentle exercise in the open air. The feeling of dragging, tension, and heat disappeared; and at the end of twelve weeks, during which the aforesaid remedies were sedulously employed, the breast was reduced almost to the natural size, and had the normal feel. The cupping-glass was now less frequently applied, and the iodine bath was used only once every ten days. The breast was then soft, nearly of the natural size, (an inch longer than the sound one,) and nothing abnormal could be felt in the interior of the gland.

Case iii. M. K., aged 16, of slender make but sound constitution, consulted Dr. Fingerhuth for a painless swelling of the right breast, of eighteen months' standing, supposed to have originated from pressure. She had tried leeches, compresses, ointments, purgatives, quackery, and homœopathy, without effect; and, when seen by Dr. Fingerhuth, the affected breast was twice as large as the sound one. She complained of fulness, tension, and flushes of heat in the breast, and stated that the tumour increased greatly in size during the menstrual periods. Her general health was good; but she menstruated irregularly, laboured under constipation, and was of a very irritable nervous habit.

With the view of removing the constipation and diminishing nervous excitement, Dr. Fingerhuth ordered aperients, iodine baths, a vegetable diet, and gentle exercise. In about a fortnight, the nervous susceptibility was so much diminished that he was able to take measures to bring on the lacteal secretion: these proved successful in the course of three weeks, and, at the end of the seventh week, the breast had diminished an inch in circumference. At the end of eleven weeks, he began to apply the cupping-glass less frequently, with the view of gradually arresting the secretion of milk; but the iodine baths and other remedial measures were continued. Under this treatment, the affected mamma was greatly reduced in size, and, though it remained somewhat larger than the left, Dr. Fingerhuth thought it advisable to suspend the further use of remedial agents, and leave the rest to nature. The patient was therefore directed to resume her usual occupations, merely taking precautions to protect the breast from pressure, or any other cause of irritation.

*Zeitschrift für die gesammte Medicin. October,* 1836.

---

*On the Forms of Lepra observed in the Russian Provinces bordering on the Baltic.* By Dr. BLOSFELD, of Riga.

DR. BLOSFELD premises his account of lepra, as observed in the neighbourhood of Riga, with some extremely just and pertinent observations on the mode in which cutaneous affections should be studied, in order to avoid the numerous sources of error which have led to so much perplexity and embarrassment in fixing the diagnosis of many forms of skin-disease. In detailing the following cases, which are intended to serve as illustrations of the various forms of lepra observed by him, he adopts the terms employed by the late Professor Struve, of Dorpat, in his "Synopsis Morborum Cutaneorum."

1. *Pes Elephantiacus,* (Lepra localis pedum of Struve.) In June, 1827, Dr. Blosfeld saw, in the hospital at Mittau, a potter's wife, aged 40, whose right lower extremity, as far as the middle of the thigh, was affected with a disease resembling elephantiasis. The diseased foot was more than double the size of the other; the toe-nails resembled claws, and grew into the flesh; the skin of the extremity was somewhat like tortoise-shell, and formed a hard, fissured, warty mass, of a dark blue colour, and exhibiting scattered points of suppuration. The patient was the only person of her family, or of the village, that laboured under the disease.

2. *Lepra localis pedum.* A Russian sailor, born in the government of Wilna, and residing in Riga for the last fifteen years, consulted Dr. Blosfeld, in January, 1836, for an affection of one of the lower extremities. The whole leg was thickened and hard, possessed little insensibility, and was white and shining. On the dorsum of the foot, above the heel, and about a hand's breadth above the ankle, three patches of moss-like excrescence, about an inch in breadth, surrounded the foot. The rest of the leg, anteriorly as far as the knee, and posteriorly to a hand's breadth above the ham, was also hard and of a dirty brown colour, bounded above by a patch of psoriasis, raised on a darker base. Over the anterior part of the tibia there was an ulcer, about a hand's breadth in diameter, painless, and covered with red, hard, warty granulations. The patient was of a gross lymphatic habit, his belly was very tense and hard, and he complained of tightness about the chest, and sense of pressure in the stomach after taking food.

The Pes Elephantiacus, with moss-like excrescences, is most frequently observed at Riga; but Dr. Blosfeld has also seen the mossy incrustation on other parts, as, for instance, the forehead.

3. *Lepra Ulcerosa.* R. G., aged 34, a merchant, born and residing at Riga, enjoyed good health up to his twentieth year, when he contracted primary symptoms of syphilis, which yielded to appropriate treatment, without any further consequences. Seven years afterwards, being at that time three years married, he was attacked with his present complaint, and was treated with mercury and various other remedies for many years, without any relief. When seen by Dr. Blosfeld, he had lost his eyelashes and eyebrows, and a portion of the hair of his head; what remained was dry and brittle. The cornea of both eyes was of the colour of mother of pearl, and covered with scurf, and the patient had been blind for two years. The nails were thickened, scabby, and partly bent backwards, partly inwards, like claws. The forehead, cheeks, chin, shoulders, and other parts of the body, were covered, partly with cicatrices of delicate white shining skin, and partly with detached or aggregated irregular spots and patches, (Morphææ,) from one to three inches in circumference, without any elevation, and of a bright copper colour. These spots were partly covered with yellowish or brownish crusts and scales, attached in the centre, but loose at the edges; the remaining portion of their surface was marked with bleeding fissures, or they were ulcerated, raw, and covered with a serous exudation. On the hands and feet, the spots and ulcerated patches were of an irregular undulating form, (Ophiasis,) from two to four lines in breadth, from a quarter to half a line in depth, and from three to five inches in length. The neighbouring uninjured portions of the skin appeared rough, dry, and without much sensibility. The patient died of marasmus, six weeks after he came under the care of Dr. Blosfeld. For some time before his death, he had fever, evidently of a hectic character, but unaccompanied by night sweats. The commencement of the disease was attended with remarkable salacity; during its progress, the patient complained frequently of indigestion and vomiting, with considerable languor and distress of respiration. His wife did not take the disease.

Dr. Blosfeld observes, that he would be inclined to range the foregoing case under the *Lepra albaras ulcerosa* of Struve, had not the tubercles been entirely absent. It appears to correspond with the description of the Lepra ulcerosa of the Greeks given by Dr. Rothamel, or to the Lepra Arabum anaisthetos of Fuchs. The following case corresponds more closely with the species described by Struve.

4. *Lepra albaras ulcerosa.* A stonemason, aged 35, who had laboured for five months under fever of the quartan type, was attacked, on the cessation of the febrile symptoms, with a peculiar eruption on various parts of his body. His forehead, cheeks, and chin were covered with warty, flattened, circular tubercles, closely aggregated, and elevated on coffee-coloured spots. Most of these had passed into the suppurative stage, and at various points the pus had dried up, and was covered with transparent yellowish crusts. Similar tubercles were found isolated on the scalp, which retained but little hair; and there were others of the same description on the legs, but without suppuration or crusts. The nostrils were swollen, brown, and uneven; and the anus surrounded by large, soft condylomata. The patient's voice was clear and natural. There was no reason to suspect that he had ever contracted syphilis, his wife was perfectly free from disease, and mercurials had proved of no benefit to him. He was of a phlegmatic and bilious temperament, slow, indolent, voracious, and lascivious; his pulse was soft and full, scarcely fifty in a minute. He was treated with bleeding and sulphureous waters, with much benefit: the tubercles disappeared, leaving behind only the coffee-coloured spots which had formed their bases. He subsequently got an attack of apoplexy, followed by epileptic convulsions and paralysis of the right side, but recovered under appropriate treatment, and is now quite free from disease.

5, 6. *Lepra Elephantiasis leonina incipiens.* Two young persons, a brother aged 16, and a sister 14, children of a potter, came under the care of Dr. Blosfeld, in September, 1826. They had been, several years previously, treated with mercurials, low diet, and various other remedies, without any benefit. The face was red and swelled; and the cheeks, forehead, and angles of the mouth covered with close-set, copper-coloured tubercles, about the size of a split hazle-nut. The nose was thickened, red, and uneven; its point sunk in and flattened. The cavity of the mouth, the throat, gums, and root of the tongue were covered with ulcers and tubercles, about the size of a millet-seed or lentil. The voice was hoarse and rough, the respiration sibilous and laboured, the muscles stiff, the skin apparently insensible, the nails thickened and scabby. Neither of them had ever contracted syphilis; and the other members of their family, as well as the rest of the inhabitants of the village, had no trace of the disease.

The foregoing cases bear considerable resemblance to Radesyge, as described by Holst, Hunefeld, and Gedike. The following is much more like *Lepra leonina,* and was recognized as such by Dr. Welz, who had seen the disease in the East.

7. The son of a Russian soldier, aged fourteen, was admitted into the military hospital at Riga, in June, 1835, having been five years ill. His forehead, cheeks, and the parts around the mouth, were beset with tubercles of a dirty copper colour: these were about the size of a split hazle-nut, more or less flattened, and closely aggregated; and, with a broad flat nose, sunk at the point, gave to the patient the appearance of a mastiff. The hair and nails were sound, the voice slightly affected from the obstruction of the nostrils. His digestion was normal In December he was attacked with typhus; during which the tubercles inflamed, scabbed, and fell off the face, leaving behind elevated patches of a dirty copper colour and husky appearance. A few rather isolated tubercles remained on the extremities. The rest of the skin was cold, moist, and clay-coloured. The patient's mother was said to have laboured under the same disease; his father and sisters were healthy. He died of marasmus in May, 1836.

8. *Lepra Crustosa,* (Psoriasis universalis alba of Struve.) Herr von H., aged 34, and residing in Curland, placed himself under Dr. Blosfeld's care in the summer of 1825. He had laboured for many years under a cutaneous affection, for which he had tried a great variety of remedies without success, and was now greatly debilitated, suffered from hypochondriasis, persistent cardialgia, constipation, and was also very salacious. With the exception of his face, palms of his hands, and soles of his feet, the whole surface of his body was covered with rough, fissured, grey crusts, so as to give him the appearance of being covered with a coat of mail. On the extremities the crusts remained unchanged, but from time to time they dropped off the trunk, leaving the bare portion of the skin moist and red: this became gradually covered with crusts, at first thin, afterwards thicker and harder. He had no pain or itching. His wife and daughter were healthy. About a year after, he died with symptoms of marasmus.

9. *Radesyge.* Louisa G., aged 27, had enjoyed good health up to her fourteenth year, when she became subject to frequent attacks of transient pain in the thigh, and irregularity of the catamenia, which appeared only once in six or eight weeks. Seven years since, she had been repeatedly exposed to cold, and, three years afterwards, she was seized with violent rending pain of the right side of the head, right ear, and right eye, accompanied by deafness and amaurosis. She had also repeatedly suffered from erysipelatous affections of the leg and face; and was attacked, in April, with fever of a tertian type, attended with great hoarseness. When seen by Dr. Blosfeld, in August, her face was swelled, red, and shining; her nose sunk in, and thickened; she was deaf on the right ear, and the right eye was amaurotic and constantly bedewed with tears. The throat and pharynx were dry and covered with red spots and ulcerated patches, the uvula was gone, the nasal bones carious, the voice hoarse and whispering, the breathing sibilous, and attended with croupy cough and paroxysms of suffocation. The pain in the head was violent, the muscles of the right side sluggish in their action, the urine turbid and exhibiting a white deposit; slow fever. Her hair and nails were sound. Although the patient never had syphilis, she had been treated for it by several physicians, but without success. The same result attended every other plan of treatment, and she left Riga in 1834.

10. *Ichthyosis Cornea.* Countess L., a Polish lady, aged 16, a blonde, of pale sallow complexion and costive habit, but otherwise healthy, was attacked with the above-mentioned affection in her third mouth. Her whole body, with the exception of the face, axilla, palms of the hands, and soles of the feet, is covered with a yellowish, rough, and wrinkled cuticle, like that of a hen's foot, particularly about the elbow and knee joints. The reticulated cuticle is beset with thin jointed scales, which fall off in great quantities in bed, and drop on the ground as she walks. This affection diminishes in summer, and increases in winter, without ever disappearing altogether. Her younger brother labours under the same disease; the rest of the family are healthy.

Dr. Blosfeld states, in conclusion, that he has frequent opportunities of observing syphilitic eruptions which bear a considerable analogy to lepra, but has omitted to give any account of them, for obvious reasons.

*Journal der Praktischen Heilkunde. September,* 1836

*On Excision of the smaller Joints.* By Dr. Gernet, of Hamburg.

Among the many improvements in modern surgery, the substitution of excision of diseased joints and of carious portions of bone for amputation of the limb, is not the least important. A considerable number of years have now elapsed since this principle was first applied to the treatment of caries of the larger joints, and, in a late Number of the Hamburg Journal, we find Dr. Gernet, assistant surgeon of the Hamburg hospital, ably advocating its extension to the treatment of caries of several of the smaller joints. He reports seven cases, which were operated upon by Fricke, in the hospital. In four of these, the caries affected the bones of the hand, and, in the remaining three, those of the foot. Of the former number, the disease in three affected the metacarpal joint of the thumb, and was produced in one case by the point of an awl penetrating the joint; in the second, by the cut of an axe; and, in the third, it was ascribed by the patient to a wire, which, some months previously, he had drawn tightly round the thumb; but the chief cause seemed to lie in the cachectic state of the constitution. In the fourth case, the caries affected the metacarpal joint of the middle finger, and, as in one of the former instances, was caused by the wound of an awl.

In those cases in which the foot was the seat of the disease, the caries affected, in the first, the joint between the first and second phalanx of the great toe, and could not be ascribed to any evident cause; in the second, no caries existed, but a large exostosis, which was attached to the head of the first phalanx of the great toe, greatly incommoded the patient. In the third case, the caries affected the metatarsal joint of the great toe.

In all of these cases, the operation was performed by removing the extremities of both bones; and this was judged the more advisable proceeding, even in the case of exostosis, where no lesion of the opposite articular surface existed. The ends of the bone were then approximated as nearly as could be done without much difficulty or causing great uneasiness to the patient, and retained in this position by a peculiar apparatus. Union by the first intention was tried in two instances, but afterwards abandoned, and the wound was stuffed with charpie. Torsion was employed to arrest the hemorrhage from the mouths of bleeding vessels; a practice which seems to be followed in all operations in the Hamburg hospital.

It has been advanced against the operation of excision of the smaller joints, that the time required for the cure, and the length and pain of the operation, were more than an equivalent for any advantage which could be derived from a shortened and anchylosed finger, and which perhaps would prove a worse than useless appendage to the patient. We shall therefore examine the results of the seven operations. Of the four cases in which the hand was affected, the success in three was complete. On an average, five and a half weeks were sufficient for the union of the wound and solidification of the bone; and all three were capable of returning to their work at the end of seven weeks. Two of them, in whom the metacarpal joint of the thumb had been affected, regained completely the use of the finger; and the other, the fourth case, was fast regaining the use of the finger when he left the hospital. In the third case, the wound healed slowly; but the patient was unable to use the thumb, and was dismissed at the end of three months, in rather an unsatisfactory state. Of the foot cases, the first was able to use the extremity at the end of five weeks; but, in the second, the cure was retarded by necrosis of a portion of bone till the end of the tenth week. Four weeks sufficed to effect the cure in the third.

In the first set of cases, the operation, including the time occupied in dressing the wound, lasted from fifteen to twenty-six minutes, the two extremes. In the second, ten, or at most fifteen, minutes were required. In no case were bad consequences, which could be ascribed to the nature of the operation, observed to follow.

*Zeitschrift für die gesammte Medicin.* Band iii. heft 4. 1836.

*On the Treatment of Varus and Pes Equinus, by the division of the Tendo Achillis.* By Dr. BOUVIER.

DR. BOUVIER presented to the Academy of Medicine (1) the extensor tendons of the foot of a dog, which had been divided, thirty days before it was killed; (2) a man, aged forty-six, whom he had cured of the pes equinus, by a division of his tendo achillis. The divided extremities in the case of the dog were united by a solid substance, which was an inch in length, consisting of a new fibrous tissue, having externally the form and appearance of the tendon itself, adhering loosely to a cellular sheath in which it was enclosed, so as to fulfil perfectly, as far as solidity and mobility were concerned, the functions of tendon. It illustrated comparatively the case of the man. The deformity of his foot had existed from infancy. The section of the tendo achillis was performed in the month of February. At the present time (October) it is difficult to ascertain the point at which the tendon was divided, although, immediately after the operation, the extremities were placed more than an inch apart, and this distance has since increased. The foot forms a right angle with the leg, the heel resting on the ground both whilst standing and walking, and the muscles of the calf act upon the calcaneum, so as to extend the foot as before the operation. The treatment lasted only fourteen days.

In a subsequent paper, Dr. Bouvier records other cases successfully treated both by himself and others. His mode of operation is as follows. The patient lies upon his belly. A slight incision is made with a lancet, parallel to the axis of the limb, a few lines in length, and at that part where the tendon is the smallest and the least prominent. Through this incision is introduced, beneath the skin, a small straight knife, with a blunt point. The tendon is then divided from without inwards, without wounding the integuments. The limb is then placed in such a way as to keep the ends of the tendon separated, the foot being flexed on the leg. The subsequent treatment is very simple. It consists in gradually increasing the flexion of the foot, until it forms an acute angle with the leg: this is accomplished in an indefinite time, according to the condition of the articular surfaces of the tarsus. If, conjointly with the elevation of the heel, the foot is turned inwards, the apparatus must be so constructed as to correct this deviation. The reunion of the tendon takes place rapidly. The cicatrix is of considerable solidity, by the fifteenth or twentieth day. Two of the six patients operated on were affected with varus; one being a young man, twenty-three years of age; the other, a female aged fifty-four. The form of the foot in each case was greatly improved. The other four as well as a patient operated on by M. Roux were completely cured. They all were affected with pes equinus.

*Bulletin de l'Academie Roy[illegible] de Medecine,* Nos. 1, 5, 6. 1836.

---

*Account of a new Instrument for plugging the posterior Nares.* By M. MARTIN SAINT ANGE.

THIS instrument (which its inventor, M. Saint Ange, names *Rhinobyon,*) consists of a straight canula of silver, five inches long, and of the diameter of a crow-quill. To one extremity is attached, by means of a circular groove, a small delicate bladder; the other, slightly belled, is furnished with a stop-cock; between this and the centre of the canula runs a slide (*curseur,*) with a projecting tongue.

In a case of epistaxis, the bladder-extremity of the instrument is passed through the affected nostril to the posterior nares; air is blown in, through the bell mouth; the cock is closed; the moveable tongue prevents the descent of the instrument into the pharynx by its pressure against the ala or septum of the nose, and is securely fixed by a screw. Air has been found preferable to cold water. A portion of intestine about three inches in length, or other similar membrane, might be made to fulfil the double indication of plugging the nares posteriorly, and of exerting pressure on the nasal walls.

*Bullet. Gener. de Therap. Med. et Chir.* 15 Janv. 1837.

*Treatment of Sprains by Friction and Shampooing.* By Dr. Maignien.

Dr. M. says, all the means hitherto employed for the treatment of these affections have had for their aim the stopping the development of inflammation, and combating it when existing; by the use of refrigerants and astringents in the first instance, and by antiphlogistics and emollients in the second. The method which he adopts was suggested to him by the homœopathic doctrines.

The treatment consists, in the first instance, of rubbing with both hands, lightly, the injured member, so as not to excite much pain. The friction is to be gradually increased in rapidity and degree. After about an hour of this treatment, some movements of the articulation are to be attempted in every direction but that in which the injury took place: this should be continued for four or five minutes. At this period much alleviation is generally experienced; the tension, heat, and swelling which existed, gradually subsiding. These means are to be continued for thirty or forty minutes longer, when the articulation is to be moved in every direction. In another fifteen or twenty minutes, the patient is to be requested to walk; and, to complete the cure, the friction and shampooing combined are to be continued two or three times on the same or following day. When the articular ligaments are broken, the success will not be so complete; nevertheless, the swelling and pain will be diminished by these means.

The following is one of the three cases reported.

M. C. fell from a wall twenty feet high, and his right hand received all the weight of his body. A violent strain, with swelling and acute pain, was the result of the accident. Dr. M. was not present, and leeches and cataplasms were applied. He ultimately, however, saw the patient, and found the member in a state of forced pronation, and stretched upon the bed, incapable of motion, without great pain. Dr. M. commenced the frictions and shampooing, and in two hours the patient could use a stick, and announced that he felt no more pain.

*Gazette Médicale de Paris. December,* 1836.

---

*Make-shift Surgery.* By Dr. Nevermann, of Mecklenburg-Schwerin.

I lately read, in the second part of Grossheim's Operative Surgery, the following description of a camp-contrivance for amputating: "In a dry spot, two parallel trenches should be dug, two and a half or three feet deep, six feet long, and two and a half distant from each other, so that the intervening space may serve as a table, the two extremities of this strip of ground must be cut through, making a clear passage all round." This recalled to my mind a lecture of Professor Fenger, of Copenhagen, delivered in 1830: "It sometimes happens in war-time," said this gentleman, "that in consequence of the numerous amputations, the instruments become quite blunt. When this occurs in a town, we may send, to a shop for some razors, fixing the blade firmly with a strip of adhesive plaster before using them. In the Camp we may employ the razor which every soldier carries in his knapsack. Much in this way," continued the professor, "Falckenthal, surgeon-in-chief of the Danish fleet, was forced to manage, after the bloody sea-fight with the English under Nelson, in the road of Copenhagen. When all his knives were rendered useless, he sent to one of the best shops for the razors that were exposed in the window; wrapping sticking plaster round the handles, he performed eighteen amputations the same night with these tools."

*Gräefe und Walther's Journ. für Chir.* xxiv. Band. iv. Heft.

---

*On the Treatment of Varicocele.* By Dr. Fricke, of Hamburg.

In varicocele, Fricke's treatment consists in drawing a thread directly through the varicose tumour. This method, he says, acts, at least in the greater number of cases, not by producing obliteration of the veins, but by exciting in them a higher contractile power, and an exudation of plastic lymph; thereby inducing a thickening of their coats, and at the same time a diminution of their caliber.

The following two cases may serve as illustrations:

CASE I. A man, aged 40, suffered from varicocele to such an extent as to produce incipient atrophy of the left testicle. Two threads were passed through the tumour, and nine days later another thread. After twenty-four days, it was found necessary to repeat the operation, when two more threads were passed, and again, in about six weeks, another. The enlarged veins did not entirely regain their natural condition, but the tumour was greatly diminished, and the testicle manifestly increased in size.

CASE II. A gentleman, aged 40, had been affected for sixteen years with swelling of the right epididymis, a consequence of gonorrhœa. Two years before applying to Dr. Fricke, he began to suffer, in consequence of an injury, from a like affection of the left epididymis, accompanied by a varicose state of the veins of the cord. A thread was drawn through the largest of the varices; the operation was followed by very slight reaction. On the fourth day, the patient ceased to make a constant use of the suspensory bandage, and two days later he gave it up entirely. Twelve days after the operation, pressure, by means of slips of adhesive plaster, was applied to the testicles; and, ten days after the commencement of this application, the patient was able to take a walk of four hours. Within four weeks from the commencement of the treatment, no trace of the varicose swelling remained; and, at the end of four months, the patient still remained free from any return of it.

*Zeitschrift für die gesammte Medicin.* Band iii. heft 4.

